# Understanding how to maintain paramedic simulation-based education quality: a qualitative study

**DOI:** 10.1186/s41077-025-00402-x

**Published:** 2026-03-10

**Authors:** Carl A. Webster, Kacper Sumera, Agnieszka Sumera, Jamie Hahn, Lawrence Hill, Helen Henderson, John Sandars

**Affiliations:** 1 Institute of Health and Allied Professions, Nottingham Trent University, Nottingham, NG11 8NS UK; 2 European Pre-hospital Research Network, Nottingham, UK; 3 East Midlands Ambulance Service NHS Trust, Education, Nottingham, NG11 8NS UK; 4 School of Health Sciences, University of East Anglia, Norwich, NR4 7TJ UK; 5 NHS Scotland Academy, Agamemnon St, Clydebank, G814DY UK; 6 School of Medicine, Edge Hill University, Ormskirk, L39 4QP UK

**Keywords:** Simulation-Based education, Paramedic education, Quality frameworks, Healthcare simulation

## Abstract

**Background:**

Simulation-Based Education (SBE) is an essential component of paramedic training, offering a structured environment for students to develop clinical skills and decision-making capabilities. Despite the existence of SBE quality standards, such as those from the Association for Simulated Practice in Healthcare (ASPiH) and the International Nursing Association for Clinical Simulation and Learning (INACSL), the application of these standards in maintaining paramedic SBE quality remains inconsistent. This study explores paramedic educators’ perceptions of SBE quality, the challenges in applying SBE quality standards, and potential strategies for improvement.

**Methods:**

An exploratory qualitative research design was employed, utilising semi-structured interviews with eight paramedic educators from five higher education institutions in the East Midlands, UK. Data were analysed using template analysis to identify key themes related to maintaining paramedic SBE quality.

**Results:**

Participants were aware of the importance of quality in SBE, yet demonstrated limited awareness and inconsistent application of formal quality standards, often relying more on informal, experience-driven approaches. Psychological safety and student engagement were emphasised as crucial to effective SBE, yet institutional constraints, including financial limitations, staffing shortages, inadequate faculty training, hindered quality assurance efforts. There were also challenges in standardising simulation scenarios and debriefing practices, with educators highlighting the need for greater institutional support and structured training in simulation pedagogy.

**Conclusions:**

The study highlights significant opportunities to enhance the integration of quality standards in paramedic SBE, emphasising the need for improved faculty training, increased institutional investment, and structured quality assurance mechanisms. Addressing these barriers is essential to ensure consistency, effectiveness, and sustainability in paramedic SBE.

**Supplementary Information:**

The online version contains supplementary material available at 10.1186/s41077-025-00402-x.

## Background

Simulation-Based Education (SBE) has become an integral component of paramedic education, providing learners with a controlled and immersive environment to develop critical clinical and decision-making skills. By replicating real-world scenarios, SBE enables students to gain hands-on experience in a safe setting, reducing risk to patients while improving competency and confidence [1]. As paramedic education increasingly incorporates SBE approaches, ensuring the quality and consistency of these simulations is paramount. To maintain high standards, various quality standards have been established to guide the development, delivery, and assessment of simulation activities. Professional bodies such as the Association for Simulated Practice in Healthcare (ASPiH) in the UK and the International Nursing Association for Clinical Simulation and Learning (INACSL) globally, have published widely recognised standards to support effective SBE implementation by maintaining its quality [2, 3]. These standards provide benchmarks for curriculum integration, faculty training, learner assessment, and scenario design, ensuring that simulation activities are pedagogically sound and aligned with learning objectives. However, despite the existence of such standards, there remains variability in how institutions and educators interpret and implement quality guidance in paramedic SBE [4]. This variability raises questions regarding the consistency and effectiveness of SBE provision across different educational settings [5]. Furthermore, while many quality standards are developed for broader healthcare education, there is limited research specifically exploring how paramedic educators engage with these guidelines and the unique challenges they face in maintaining SBE quality [5].

While quality standards provide structured guidance, their successful implementation in paramedic SBE is not without challenges. Firstly, resource availability is a significant factor. Effective simulation requires specialised equipment, trained faculty, and sufficient time allocation within the curriculum, all of which can be constrained by institutional budgets and competing educational priorities [6].

Secondly, educator preparedness and training is essential to ensure quality SBE. While many paramedic educators have extensive clinical experience, they may not have received formal training in simulation pedagogy [7, 8]. This can result in inconsistent debriefing practices, ineffective scenario design, and varied approaches to student assessment, all of which may impact the learning experience if not meeting the standards required [9]. Ensuring educators are well-trained in simulation best practices is essential for maintaining quality. Frameworks provide an opportunity to standardise practice and enhance SBE provision, while supporting educators in developing awareness and understanding of such approaches can further strengthen and sustain quality.

Institutional support and policy alignment may also be a barrier to high-quality SBE. Universities and training providers may have different interpretations of quality standards, leading to inconsistencies in adherence and application [5]. Without clear institutional policies that align with national or international standards, paramedic educators will struggle to implement best practices effectively [10, 11]. Balancing SBE with traditional teaching methods and clinical placements poses logistical and pedagogical challenges, particularly when simulation is viewed as an adjunct rather than an essential component of training. While frameworks and standards provide recommendations for evaluating SBE, there is no universal approach to measuring its effectiveness in paramedic SBE.

Simulation is a complex social endeavour making the determination of appropriate, quantifiable metrics for student learning outcomes, faculty performance, and perceptions of simulation fidelity particularly challenging [12]. This is in part because “fidelity” - *faithfulness to the fiction contract* [13] - emerges where simulation design curates physical/environmental, conceptual, and psychological aspects of experience in a manner that is phenomenologically authentic to the learner/participant [12, 14]. Quantifying phenomenological features of experience is challenging and arguably philosophically incongruous. While studies have demonstrated that qualitative non-technical skills such as situational awareness and decision-making can be evaluated using structured approaches [15], objective measures of SBE quality remain less well developed. Furthermore, the inherently dynamic and context-specific nature of SBE complicates efforts to establish universally applicable evaluation frameworks. Greater emphasis on refining reliable and valid assessment tools, as well as integrating both qualitative and quantitative measures, may support a more comprehensive understanding of SBE effectiveness. Without a robust mechanism for assessing quality, ensuring consistent improvement in SBE provision remains difficult. Despite the growing emphasis on SBE in paramedic education, limited research has explored how paramedic educators understand, interpret, and implement SBE quality standards in practice [4]. Much of the existing literature on SBE focuses on nursing and medical education, with fewer studies examining its role within paramedic training specifically [5]. While general healthcare simulation guidelines exist, their applicability and effectiveness in the paramedic context remain underexplored.

Given these gaps, the primary aim of this study was to explore paramedic educators’ perceptions of SBE quality, the challenges in applying SBE quality standards, and potential strategies for improvement. Specifically, the research seeks to answer the following questions:

- How do paramedic educators define and understand quality in SBE.
- What are the perceived challenges in implementing and adhering to existing SBE quality frameworks?
- What strategies do educators identify to enhance the quality and consistency of SBE?

To address these questions, this study employed a qualitative research design using semi-structured interviews with paramedic educators. This approach facilitated an in-depth exploration of their experiences, insights, and recommendations, providing a nuanced understanding of SBE quality from those directly involved in its delivery. The study acknowledges both existing challenges—such as the lack of evidence and inconsistencies in practice—and opportunities for collective self-assessment, fostering a collaborative approach to overcoming barriers and enhancing SBE provision.

## Methods

### Study design and research method

An exploratory qualitative approach was conducted using semi-structured interviews. The study was approved by the Schools of Business, Law and Social Sciences Research Ethics Committee at Nottingham Trent University (REC application ID: 1844104).

### Sampling and setting

Recruitment was by email invitation to simulation leads and facilitators at higher education institutions in the East Midlands, United Kingdom delivering paramedic SBE. All participants were provided with an information sheet. Inclusion criteria were current involvement in the design, facilitation, or leadership of paramedic SBE.

### Data collection and analysis

Recruitment and interviews were conducted between December 2024 and May 2025. Following informed consent, all interviews were conducted with one participant at the time via Microsoft Teams (version 24295.605.3225.8804, November 2024; Microsoft Corporation) by an experienced qualitative researcher (JH) and an SBE expert (AS). Each interview lasted between 30 and 75 min. Microsoft Teams was also used to audio-record and generate an initial transcription of each interview. The interview guide, included in Additional File 1, was refined following a pilot, which highlighted the need to adapt questions based on participants’ familiarity with and use of relevant quality frameworks or standards. The questions explored perceptions of quality, challenges in adhering to SBE quality standards, and strategies for enhancing quality in SBE. The anonymised transcripts were reviewed for accuracy before being analysed using template analysis [16], which offered both flexibility and structure in exploring participants’ perspectives. Analysis began with immersion in the data through repeated readings of the transcripts. Initial coding was conducted independently by two researchers (CW, KS), with codes reflecting both a priori interests (e.g., perceptions of quality, awareness of standards) and inductive insights emerging from the interviews. These codes were organised into a provisional coding template that was iteratively refined through comparison across transcripts. Related codes were then clustered into higher-order themes, which were reviewed and refined through discussion within the research team. Consensus on the final thematic structure was achieved through collaborative review of illustrative extracts, with oversight from a senior qualitative researcher (JS) to enhance credibility. Reflexivity was maintained throughout, with the team acknowledging how their professional backgrounds, as paramedic educators, clinicians, and qualitative researchers, influenced both the coding process and interpretation of findings. Member checking was not undertaken due to anticipated low engagement within the study’s time constraints, as well as ongoing debate regarding its contribution to data trustworthiness [17].

## Results

Eight participants took part in the study, representing five higher education institutions delivering paramedic SBE in the East Midlands. Two participants were senior academic leaders involved in strategic decision making, both with substantial experience in the design and delivery of SBE. The remaining participants had between one and ten years’ experience of integrating simulation into the paramedic curriculum. Their professional and educational backgrounds varied: some held master’s-level qualifications in leadership and education, while others held undergraduate degrees in paramedic science and were working towards postgraduate teaching qualifications, such as a PGCert in education.

### Frameworks and standards for SBE

Many participants reported not relying on formal frameworks or standards to guide their SBE practices. For example:

*We base it around the wider learning objectives and fit it to whoever the learners are* [participant 4].

Some of the participants were either unaware of formal frameworks for assessing the quality of SBE or could not recall them:

*Being honest*,* I wouldn’t know of any* [participant 1]

*I wouldn’t know what they were called … I’ve googled things and I’m sure that’s the sort of thing that has come up* [participant 2].

Instead, informal methods, shaped by personal or team experiences, were commonly used:

*…we seek informal feedback quite a lot. Particularly with the simulation afterwards* [participant 6].

*Probably not a formal tool. Obviously we measure quality based on our student feedback*,* primarily in the sense of whether they’re engaging with it*,* whether their attendance is good initially is that kind of bit of benchmarking* [participant 1].

Some participants were making greater efforts to align their practices with established frameworks or standards such as ASPiH and INACSL. Those who did so benefited from the guidance of a simulation expert and the growing integration of quality frameworks:

*The university employed like a simulation lead… they’ve helped kind of like align a framework for the university to kind of work towards* [participant 3].

*Overall*,* we’ve just generally improved our simulation experience* [participant 6].

However, despite the widespread adoption of these standards, there were persistent concerns regarding their applicability to paramedic SBE and also the adequacy of SBE as a substitute for clinical practice.

*I appreciate that they’re in a way more nursing based* [participant 3].

*…maybe not as applicable for the paramedic side of things* [participant 3]

*Won’t replace practice. You know it’s being held as this thing that like if you don’t get 400 h of clinical practice*,* you can do 100 h in simulation it might*,* it might make you more competent*,* but it won’t replace the environmental factors* [participant 8].

#### Essential factors for ensuring quality

Psychological safety and student engagement were identified as essential for effective SBE. Most participants emphasised the need to create supportive environments where learners felt comfortable making mistakes without fear of judgment:

*But how do you look to engage everybody else and that’s something that we’ve really started…I want them to feel safe. So I want them to know that it’s a safe environment that they can make mistakes*,* that there’s no judgement that they can*,* you know*,* be involved in this in this process* [participant 1].

*I think truly effective simulation does involve you making the people that involved in it feel safe* [participant 4].

*Simulations themselves are very safe. They’re very inclusive. You know*,* they are psychologically safe* [participant 6].

*I think that it’s a really good educational tool because it’s safe. But actually there are elements of that that need to be exposed to the students need to be exposed to in a controlled manner face to face* [participant 7].

Adequate time, staffing, and physical resources were consistently identified as essential but an ongoing barrier to delivering quality SBE:

*Yes*,* the time and the people needed because it seems to be really high…and how do you fund that day?…how do you justify it? How do you justify having that amount of people that are needed?* [participant 1]

*Invariably*,* because the resource pressures I don’t I end up kind of running one of the sims myself*…*There’s nothing worse than telling learners they’ll have time*,* and then rushing because you’re running out of it* [participant 4].

*Finance manning some of these simulation days do take a lot of manning and all that*,* and with the constraints that universities are under*,* it’s more about justifying why* [participant 5].

*I think the main challenges are not really related to the standards*,* it is just related to all the external factors that drive into things like trying to increase cohort sizes*,* trying to do everything for less money* [participant 6].

*I think the tech frightens people…Time pressure is the big one*,* and obviously numbers* [participant 7].

#### Consistency across sessions

Consistency in SBE scenarios was identified as important for quality assurance and was often maintained through informal methods, such as templates, predefined learning objectives, and peer feedback. Regular feedback from students and team discussions were cited as crucial for identifying inconsistencies and improving future sessions:

*I have a clinical scenario template which I’ve developed based on kind of previous experience that provides structure and utilises learning outcomes specific learning objectives* [participant 1].

*…also try and map out the kind of clinical course of that scenario where we see that going. Kind of list of key learning points so that everybody is getting that kind of summary key learning points* [participant 7].

*In watching colleagues do that very well and try to implement to do that bit more structured as well* [participant 4].

Scaffolded learning objectives and the involvement of senior students in mentoring roles were also used as strategies to standardise quality across sessions:

*We gather feedback every time to see if anything needs to be changed moving forward*. *We use third-year students to mentor lower groups*,* ensuring consistency across levels* [participant 5].

#### Challenges in designing and implementing simulation

Budget limitations were a common challenge, particularly when acquiring high-technology mannequins and maintaining advanced simulation technology. These constraints impacted the ability to deliver consistent, high-quality simulations:

*What I’d like and what we can afford are very different things* [participant 5].

Facilitators often lacked formal training in simulation design and delivery, leading to variability in session quality:

*Some people struggle with the tech*,* and it impacts their ability to run scenarios effectively* [Participant 7].

A lack of strategic institutional support and clear direction for simulation-based education was identified as a significant barrier:

*The organisation lacks a strategic direction—there isn’t one* [participant 8].

#### Use of informal guidelines and best practice

Structured debriefing models, such as PEARLS and TeamGAINS, were widely used to enhance learning outcomes. However, their application was inconsistent across participants:

*Debriefing is vital*,* but it’s not always done in the same way* [participant 6].

*I’ve certainly followed other frameworks for the debrief section of simulation* [participant 4].

*…definitely like debriefing models*,* say things like pearls or team gains*,* you know*,* debriefing tools like that definitely* [participant 1].

Informal sharing of best practices among facilitators and drawing on professional experiences were seen as central to improving simulation quality:

*We rely a lot on what we’ve learnt from our own experiences* [participant 3].

Simulations involving various healthcare roles and input from service users were noted as effective in enhancing realism and relevance:

*We involve service users to make the scenarios more realistic and engaging* [participant 5].

## Discussion

The study had the aim of exploring paramedic educators’ perceptions of SBE quality, the challenges in applying SBE quality frameworks or standards, and potential strategies for improvement. The findings provide insights into each of these areas and highlight key implications for future practice.

### How do paramedic educators define and understand quality in SBE?

Paramedic educators identified psychological safety, student engagement, and consistent scenario delivery as key factors influencing quality in SBE. This aligns with established quality standards, such as those established by the INACSL, which emphasise the need for a standardised, consistent, and equitable approach to SBE [18]. Such an approach, supported by faculty proficient in scenario design, facilitation, and debriefing, is essential for developing resilient, compassionate, and safe practitioners who meet professional standards. Participants underscored the importance of creating a safe learning environment where students feel comfortable making mistakes without fear of judgment. This aligns with existing literature that emphasises psychological safety as a fundamental element of effective simulation-based learning that can be promoted through effective pre- and de- briefing [18]. Additionally, student engagement was identified as a crucial factor, with scaffolded learning, peer mentoring, and structured debriefing seen as strategies to enhance learning outcomes. However, inconsistencies in debriefing practices suggest a need for greater adherence to established models such as PEARLS or TeamGAINS [19].

Despite the existence of structured frameworks for quality assurance in SBE, as well as standards developed by professional bodies such as ASPiH and INACSL, many educators reported limited awareness or reliance on these resources. Instead, they tended to use informal, experience-driven methods to determine SBE quality. This suggests that while educators recognise key elements of high-quality SBE, there is a lack of standardisation in how quality is defined and implemented. Notably, examples of good practice exist even among those who have not received optimal training and development, highlighting the role of individual expertise and experience in shaping effective SBE delivery.

### What are the perceived challenges in implementing and adhering to existing SBE quality frameworks?

Participants identified significant barriers to implementing formal SBE quality frameworks and standards in paramedic SBE. A primary challenge was insufficient institutional support, specifically in financial and human resources. Educators frequently felt compelled to justify the cost of SBE, reflecting a long-standing concern about its cost-benefit and cost-effectiveness [20–23]. Cost is a major barrier to the implementation of quality SBE in healthcare [24, 25]. The costs of equipment purchase, training materials, equipment maintenance, staffing, and facility costs can be prohibitive for many institutions [23]. This mirrors the results from paramedic educators where many paramedic programs do not have access to the resources they need to provide high-quality SBE for their students. These limitations not only impact the quality of simulation but also hinder educators’ ability to fully adhere to recognised standards [23, 26]. Other professions have explored different staff roles, including voluntary positions, to establish a more cost-effective SBE provision [27]. While managing large student numbers can be challenging [28] incorporating observer roles can enhance learning outcomes and student satisfaction, particularly when structured observer tools are utilised [29]. These challenges are further compounded by institutional staff-student ratio policies, which often fail to account for the increased teaching effort required in health sciences programmes. Unlike other disciplines, health science programmes must meet the demands of professional, statutory, and regulatory body (PSRB) requirements, with SBE being a resource-intensive teaching modality. This misalignment in staff-student ratio expectations can hinder the effective implementation of SBE, limiting its potential benefits for student learning and clinical preparedness [30].

High-technology equipment and advanced facilities are often erroneously conflated with the concept of high-fidelity [31], yet fidelity describes authenticity that extends beyond technology to encompass psychological, environmental, and conceptual aspects of the learner or participant experience [31]. Evidence from international initiatives such as the Helping Babies Breathe programme in Tanzania demonstrates that low-tech manikins and simple, context-appropriate training materials can still yield significant educational and clinical impact [32]. This highlights the potential value of lower-cost approaches in contexts where resources are constrained – arguably the lowest level of technology required to achieve faithfulness to the fiction contract is the correct level of technology. Similarly, models such as those used at the Copenhagen Academy for Medical Education and Simulation (CAMES), where medical students act as simulation educators and support faculty, demonstrate innovative ways to expand faculty capacity and embed peer-led learning [33]. This aligns with Hamstra et al. [34], who argue that equating fidelity with technological mimicry is misguided, and advocate instead for forms of realism—psychological, functional, conceptual—that are educationally relevant.

Another challenge was the lack of formal training in simulation pedagogy. Many educators reported that they had received little to no structured training in simulation design and delivery, leading to inconsistencies in scenario execution and debriefing methods. This finding aligns with previous research that emphasises faculty development as a critical component of high-quality SBE [35, 36]. Without targeted training, educators may struggle to effectively integrate established frameworks into their teaching practice. Additionally, while many participants valued consistency in simulation delivery, they largely relied on informal strategies such as templates, predefined learning objectives, and peer feedback rather than structured quality assurance mechanisms. Free, open-access training resources, including simulation-focused podcasts and online modules, are available and provide accessible opportunities for professional development [13, 37]. However, the formal evaluation of their efficacy and value remains limited. This suggests that, while efforts are being made to maintain standards, the absence of formalised processes and robust assessments of training resources may contribute to variability in SBE delivery.

### What strategies do educators identify to enhance the quality and consistency of SBE?

Participants recognised the need for high quality SBE in paramedic education and, despite not recognising formal frameworks or standards, many components and strategies to improve the quality and standardisation of SBE where identified. A key recommendation was the need for structured faculty training to enhance educators’ confidence and capability in delivering high-quality SBE [38]. By embedding simulation pedagogy within professional development programmes, institutions can ensure that educators are equipped to implement best practices effectively, beyond basic learning outcomes [38, 39]. Additionally, the integration of established guidelines, such as the ‘12 Tips for the Pre-Brief’ [40], offers a practical framework to support simulation delivery and could serve as a valuable metric for evaluating SBE quality. Another important strategy was the establishment of formal quality assurance mechanisms, such as peer review processes and simulation accreditation, to support consistency in delivery [41]. While educators currently rely on informal approaches, a more structured framework could provide clearer guidance and enhance standardisation across simulation sessions. However, given that SBE in healthcare education is nuanced with multifaceted social interactions, expecting a high degree of homogeneity between sessions may be unrealistic [12]. Instead, evaluation efforts should focus on assessing the hallmarks of effective SBE, including comprehensive pre-briefing, the application of appropriate simulation pedagogy, and reflective debriefing practices using techniques such as advocacy with inquiry [42]. By targeting these core elements, institutions can foster a shared understanding of quality without constraining the adaptive and context-specific nature of SBE. Financial investment was also highlighted as a necessary step in improving SBE quality, and clearer institutional strategies and policies are required to support the long-term integration of high-quality SBE within paramedic education.

### Implications and future directions

The findings of this study have important implications for practice and policy in SBE within paramedic education. For educators, the results highlight the need for structured faculty development programmes to enhance confidence and consistency in SBE delivery. Ensuring that educators receive formal training in simulation pedagogy can improve adherence to established quality frameworks and enhance student learning experiences. For managers and institutional leaders, the study underscores the necessity of sustained investment in simulation resources, including equipment, staffing, and faculty training, to support high-quality SBE provision. Without adequate institutional support, the ability to implement standardised, high-quality SBE is significantly hindered; and yet could provide wider institutional advantages [43, 44].

At a national level, professional bodies and accreditation organisations should consider integrating SBE quality frameworks and standards into paramedic education standards, ensuring that best practices are embedded within curricula [45]. Establishing clearer policies and accreditation requirements for SBE would help standardise simulation practices across institutions, promoting equitable learning experiences for paramedic students and improving overall professional preparedness. Currently, the unregulated nature of SBE in comparison to both the health and education sectors presents a challenge. While clinical practice is governed by stringent professional standards and educational programmes undergo rigorous quality assurance processes, SBE often lacks equivalent oversight. A potential model for strengthening quality assurance in UK SBE can be drawn from the more rigorous accreditation processes used in the United States (US) where simulation centres and programmes are expected to obtain formal accreditation through organisations, such as the Society for Simulation in Healthcare (SSH), which assess against detailed standards in core areas including education, assessment, research, and human simulation. These standards, underpinned by the INACSL standards, require clear evidence of quality in simulation delivery and institutional commitment to best practice. Accreditation is widely expected for institutions delivering SBE in the US, contributing to a more consistent and regulated national approach [2]. ASPiH has developed UK-based quality standards and introduced an accreditation process designed to be inclusive and accessible [3]. As this programme continues to evolve, there are opportunities for the UK context to learn from and adapt internationally recognised models of best practice, ensuring alignment with established quality standards while also reflecting local needs. Strengthening such alignment may support the development of high-quality SBE provision across paramedic education, encompassing a wide range of simulation modalities, that contribute to advancing educational quality and the evidence base for best practice in service delivery. Introducing greater regulatory guidance for simulation in paramedic education will enhance consistency, drive continuous improvement, and ensure high-quality learning experiences across institutions.

## Conclusion

While quality frameworks and standards exist to guide SBE, their adoption within paramedic SBE remains inconsistent. The findings highlight the importance of psychological safety, student engagement, and institutional support in delivering high-quality SBE. Addressing barriers related to resource constraints and educator training is crucial for ensuring that paramedic students receive effective and standardised simulation experiences. By implementing structured faculty development, increasing institutional investment, and refining quality assurance mechanisms, the provision of SBE can be enhanced to better meet the needs of paramedic education.

### Limitations

A key strength of this study is its contribution to an underexplored area of paramedic education, offering one of the first qualitative investigations into paramedic educators’ perceptions of SBE quality. By gathering insights from multiple institutions, the study provides a broad perspective on shared challenges and best practices, enhancing its relevance to the wider paramedic education community. Additionally, the use of semi-structured interviews allowed for an in-depth exploration of educators’ experiences, capturing nuanced perspectives that may not be evident in quantitative research. However, there are several limitations of the study. First, the limited awareness of SBE quality frameworks by some educators may have reduced their stated use of approaches for ensuring the quality of the SBE that they provided and also there may have been limited experience of using low resource SBE in the UK context. Second, the focus of the study was on a single region of the UK, which may restrict the generalisability of findings to other geographical contexts. Future research should aim to include a more diverse sample across different regions and international settings to gain a more comprehensive understanding of SBE quality in paramedic education. An important aspect of future research will be to consider an understanding of SBE quality when low resource SBE is implemented, especially international settings that include Low Income Countries and Low and Middle Income Countries. Exploring these alternative models represents an important avenue for future inquiry, particularly in both UK and international contexts where resources may be limited. Furthermore, while the study highlights key barriers and enablers of high-quality SBE, further investigation is needed to explore how institutional policies and national guidelines influence SBE implementation across different educational settings.

## Supplementary Information

Supplementary Material 1.

## Data Availability

In line with the ethics approval, full transcripts are not shared beyond the research team. However, excerpts of the de-identified transcripts are available on request. Additional file 1 contains the interview guide.

## Abbreviations

ASPiH: Association for Simulated Practice in Healthcare
INACSL: International Nursing Association for Clinical Simulation and Learning
PEARLS: Promoting Excellence And Reflective Learning in Simulation
REC: Research Ethics Committee
SBE: Simulation-Based Education
SSH: Society for Simulation in Healthcare
TeamGAINS: Team-Gather, Analyse, Integrate, Narrow, Summarise (structured debriefing model)
UK: United Kingdom
US: United States
