# Supplementary material for: Understanding how to maintain paramedic simulation-based education quality: a qualitative study
Source: Adv Simul (Lond). 2026 Mar 10;11:40. doi: 10.1186/s41077-025-00402-x (PMC13185405; doi:10.1186/s41077-025-00402-x)
Supplement: Supplementary file 1 — Supplementary Material 1. [file 41077_2025_402_MOESM1_ESM.docx]

SES2 Interview Prompt Sheet

## Introduction

- Introduce yourself
- Confirm consent to record
- Confirm that they have had a chance to review information leaflet (if not, allow them to)
- Confirm eligibility criteria and consent to continue, reiterate that they can stop the interview at any time

Gain consent by asking them to confirm the following:

- I confirm that the purpose of the research has been explained to me, that I have been given information about it in writing, and that I have had the opportunity to ask questions about the research.
- I understand my part in the research. I understand that my participation is voluntary, and that I am free to withdraw [details to be inserted here], without giving any reasons.
- I understand and consent to the methods of data collection and particularly to any electronic recording that will take place and confirm that I know how long their data will be retained.
- I understand any limits to confidentiality you may need to impose and agree to any sharing of data that is proposed with people outside the project team.
- I give permission for quotes from the interview to be included in research outputs.
- I give permission for the anonymised interview transcripts (which will not make reference to any specific individuals or events) to be made available via the Open Science Framework data archive
- I agree to take part in this project.
- I confirm that I am 18 years of age or older.

## Interview Prompts:

**Understanding of Quality**

‘When you provide SBE – do you use any framework or standards for ensuring the quality?’

**IF YES TO USING STANDARDS**

"Can you describe the framework or approach you use to ensure high quality in SBE?"

"How did you decide on this particular framework or approach to ensure high quality in SBE?"

*Probe to be specific about which components or aspects of the framework or approach that they liked or not liked, and why*

"What specific standards do you adhere to in providing simulation-based education?"

"How essential do you consider these standards are for ensuring high quality in SBE?"

*Probe to be specific about why these standards are essential?*

"Can you share an example of how the chosen framework or approach has directly impacted on the quality of an SBE session?"

*Probe to be specific about which components or aspects of the framework or approach that they have used and not used, and why*

"In what ways do you monitor and evaluate the effectiveness of this framework or approach in ensuring high quality in SBE?"

*Probe to be specific about what ways they monitor and evaluate*

**IF NO TO USING STANDARDS**

"When developing a simulation session, what factors or elements do you consider essential for ensuring it is of high quality?"

*Probe to be specific about which factors or elements they choose, and why*

"How do you ensure consistency in quality across different simulation sessions without a formal framework?"

*Probe to be specific about how*

“Are you aware of any formal frameworks to ensure high quality in SBE?”

*Probe to be specific about which formal frameworks*

*If aware, probe why they are not using these frameworks*

"Do you utilise any informal guidelines or best practices to decide the high quality of your SBE sessions?"

*Probe to be specific about which guidelines or best practices used, and why*

"Do you see a potential role for adopting a standard or framework to improve the quality of SBE in the future?"

*Probe to be specific, and why*

**Ease and Challenges of Adherence:**

**IF YES TO USING STANDARDS**

"Are there any specific challenges you face in adhering to these standards? What are the challenges you face?"

*Probe to be specific about each challenge, and why*

"How do you address and overcome these challenges to ensure adherence to standards?"

*Probe to be specific for each challenge, and why*

"Have you identified any resources or support mechanisms that have been particularly helpful in adhering to these standards?"

*Probe to be specific, and why*

"Are there any standards or aspects of the framework you find particularly easy to adhere to and implement? Why?"

*Probe to be specific, and why*

“Are there any standards that stand out as particularly straightforward?”

*Probe to be specific, and why*

**IF NO TO USING STANDARDS**

“Are there any challenges you find to designing and implementing high quality SBE?”

*Probe to be specific for each challenge, and why*

“What aspects of SBE do you find easy to implement? Are there any elements that stand out as particularly straightforward?”

*Probe to be specific for each aspect, and why*

"What measures have you put in place to maintain the quality of SBE without the formal adherence to specific standards?"

*Probe to be specific for each measure, and why*

**Improving Standards:**

**IF YES TO USING STANDARDS**

“Do you have any suggestions on how the standards you use could be improved?”

*Probe to be specific for each suggestion, and why suggested*

“In what format would you prefer the standard or framework to be provided?”

*Probe to be specific about the preference for each format, and why*

"How do you envision the evolution of these standards to better meet the needs of ensuring the high quality of SBE in the future?"

*Probe to be specific for each suggestion, and why each chosen*

**IF NO TO USING STANDARDS**

‘If you are not using standards, do you see a role for a standard or framework in improving the quality of SBE?’

*Probe to be specific for each reason, and why*

"What key elements would you prioritise in the development of a new standard or framework for SBE?"

*Probe to be specific about each key element that included, and why*

“In what format would you find accessible in a standard or framework to be provided?”

*Probe to be specific about the preference for each format, and why*

## Closing Thoughts

**Additional Insights:**

“Are there any other insights or comments you’d like to share about your experiences or perspectives on simulation-based education in paramedic training?”

## End of Interview
